# Supplementary material for: Simulations suggest a constrictive force is required for Gram-negative bacterial cell division
Source: Nat Commun. 2019 Mar 19;10:1259. doi: 10.1038/s41467-019-09264-0 (PMC6425016; doi:10.1038/s41467-019-09264-0)
Supplement: Supplementary file 1 — Supplementary Information [file 41467_2019_9264_MOESM1_ESM.pdf]

Simulations suggest a constrictive force is required for Gram-negative bacterial cell division

Nguyen et al.

Supplementary Information

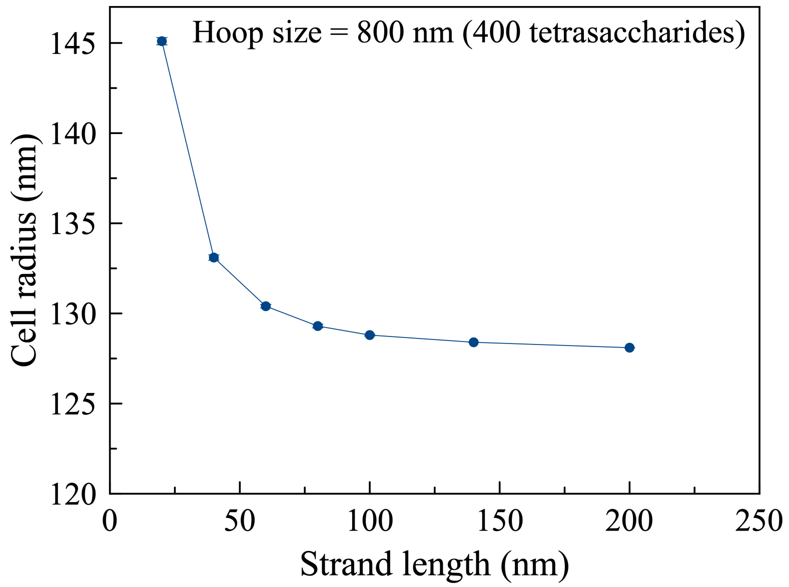

Supplementary Figure 1: Radius of the cell wall cylinder under a turgor pressure of 3 atm. The cell wall was made of hoops with the hoop size of 400 tetrasaccharides. Increasing the average PG length reduced the number of terminal crosslinks therefore reducing the cell wall's radius.

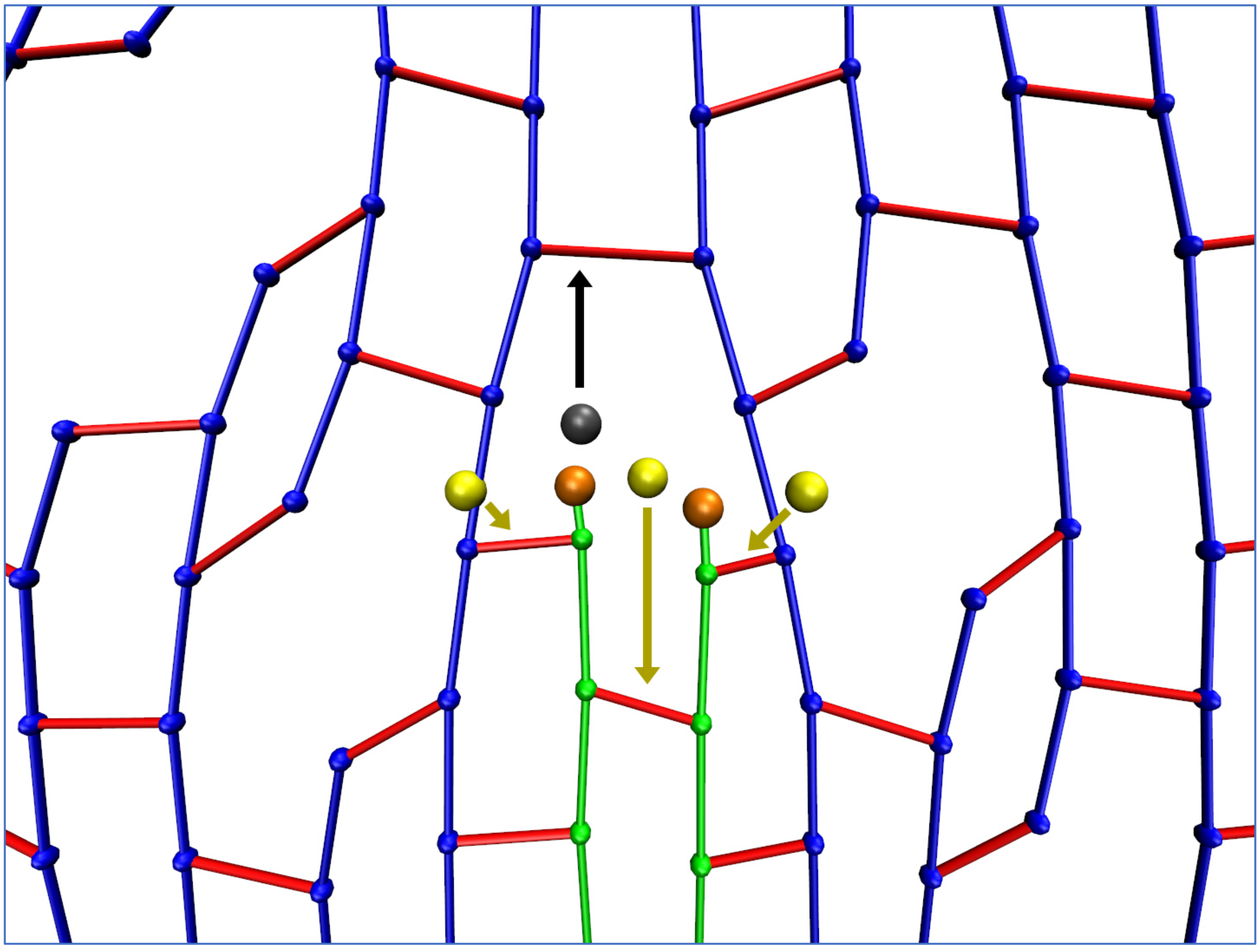

Supplementary Figure 2: Composition of a PG remodeling complex. Two transglycosylases (orange) synthesize two new glycan strands (green) at a time. Three transpeptidases (yellow) form three rows of crosslinks (indicated by yellow arrows), one connecting the two new strands to one another, one connecting the left new strand to the network, and the other connecting the right new strand to the network. One endopeptidase (black) cleaves an upstream peptide crosslink (indicated by the black arrow), releasing two peptides available for transpeptidation. Existing glycan strands are in blue, peptide crosslinks in red.

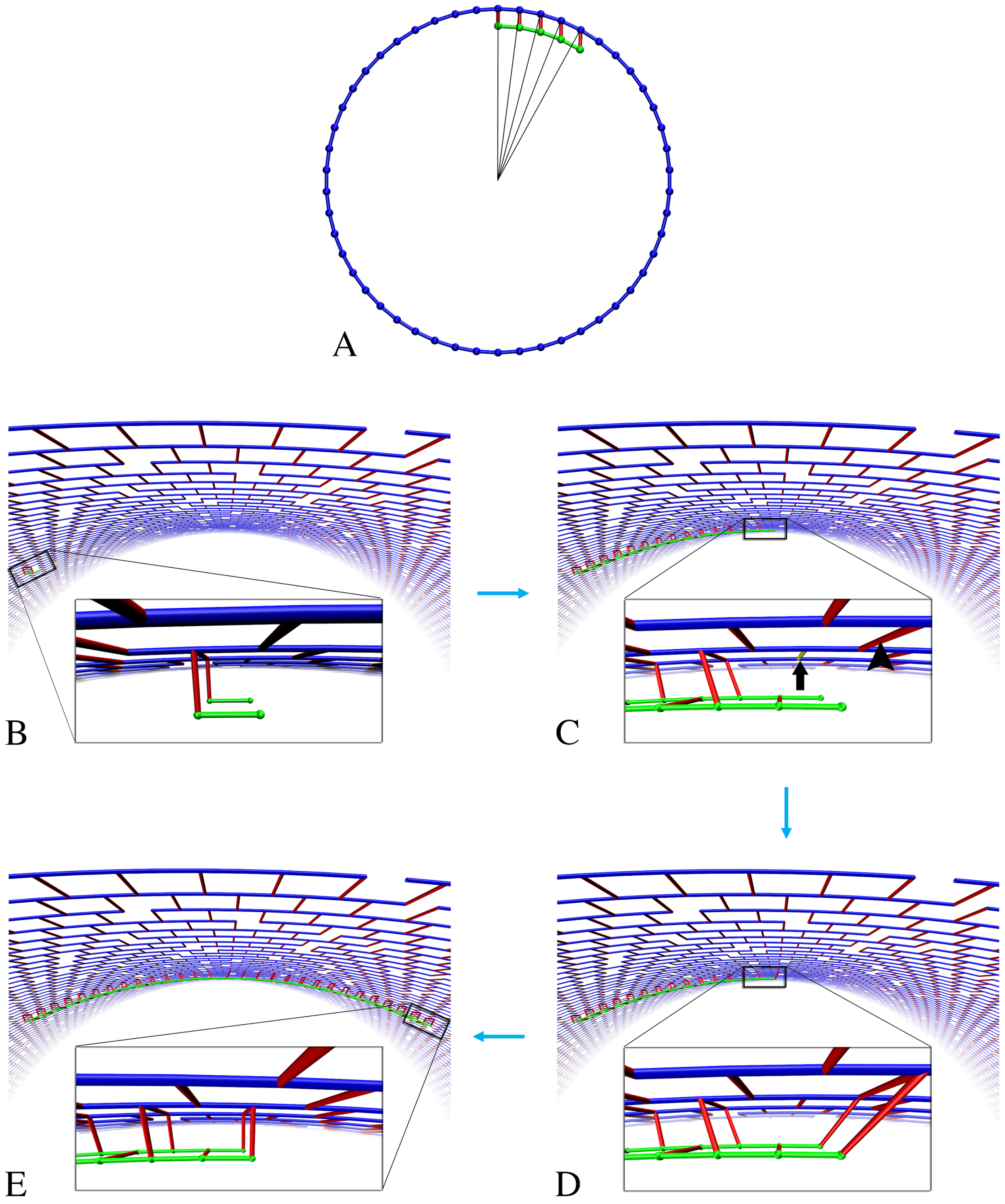

Supplementary Figure 3: A conceptual model of the make-before-break mechanism in an unpressurized cell. (A) A 2D view showing that if the first bead on the inside hoop is in register with its template on the outside hoop, subsequent beads get farther and farther ahead of their templates. Note that, for clarity, objects are not drawn to scale. (B) A 3D view showing that the

first two beads on the two new hoops are in register with their template on the existing network (we assumed new glycan strands are synthesized in pairs). (C) After ~ 30 pairs, the new strand tips are sufficiently ahead of the default template peptide crosslink (yellow, indicated by arrow) that they are closer to a more forward crosslink on the adjacent track (arrowhead). (D) As a result, the default template is skipped and new strand tips are crosslinked to the new template, which is now ahead of the tips. (E) After another ~30 pairs, the tips catch up and are in register with the template again. Template skipping therefore occurs every ~ 60 pairs of beads. Note that cyan arrows indicate the time sequence of events.

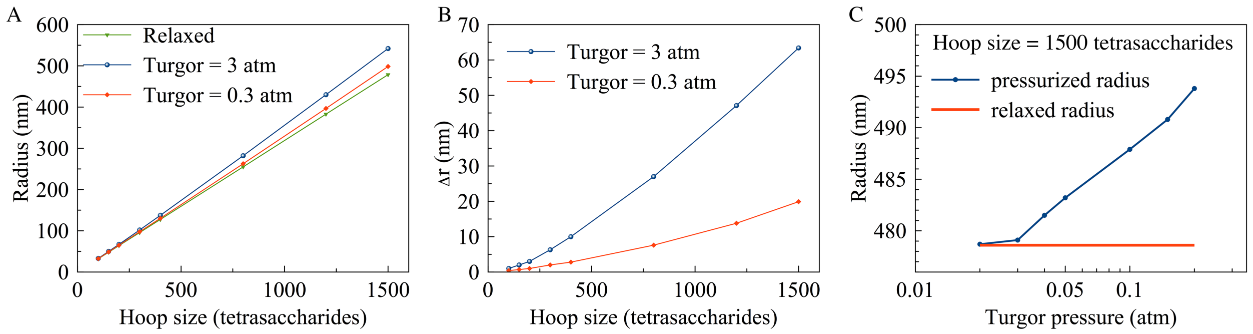

Supplementary Figure 4: Cell wall expansion under turgor pressure. (A) Relaxed and pressurized radii of the starting PG cylinder as functions of the hoop size. (B) Difference between the relaxed and pressurized radii as functions of the hoop size. (C) Pressurized radius as a function of turgor pressure of the starting PG cylinder with a hoop size = 1500 tetrasaccharides. The red line indicates the relaxed radius for reference.

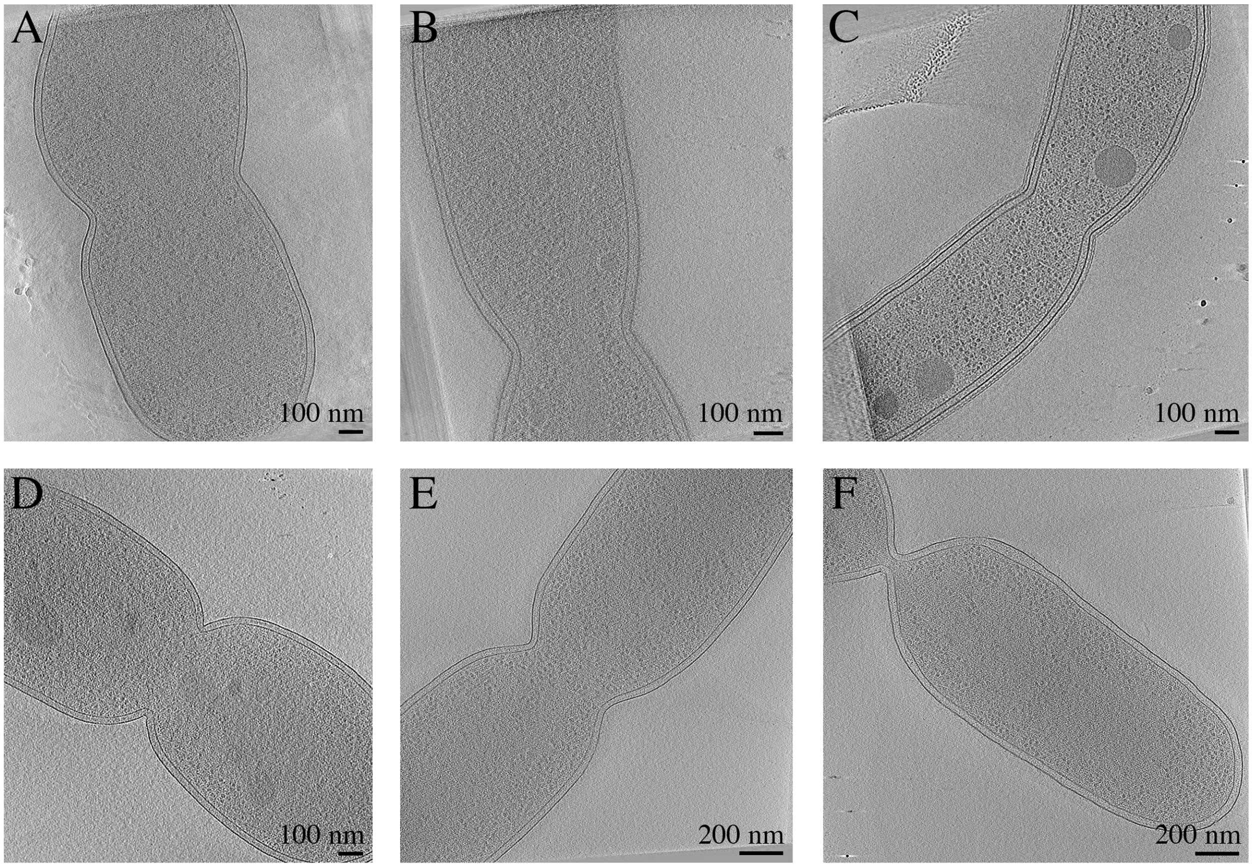

Supplementary Figure 5: Tomographic slices through dividing Gram-negative bacteria: (A) *Proteus mirabilis*, (B) *Myxococcus xanthus*, (C) *Caulobacter crescentus*, (D) *Cupriavidus necator*, (E) & (F) *Shewanella oneidensis*.

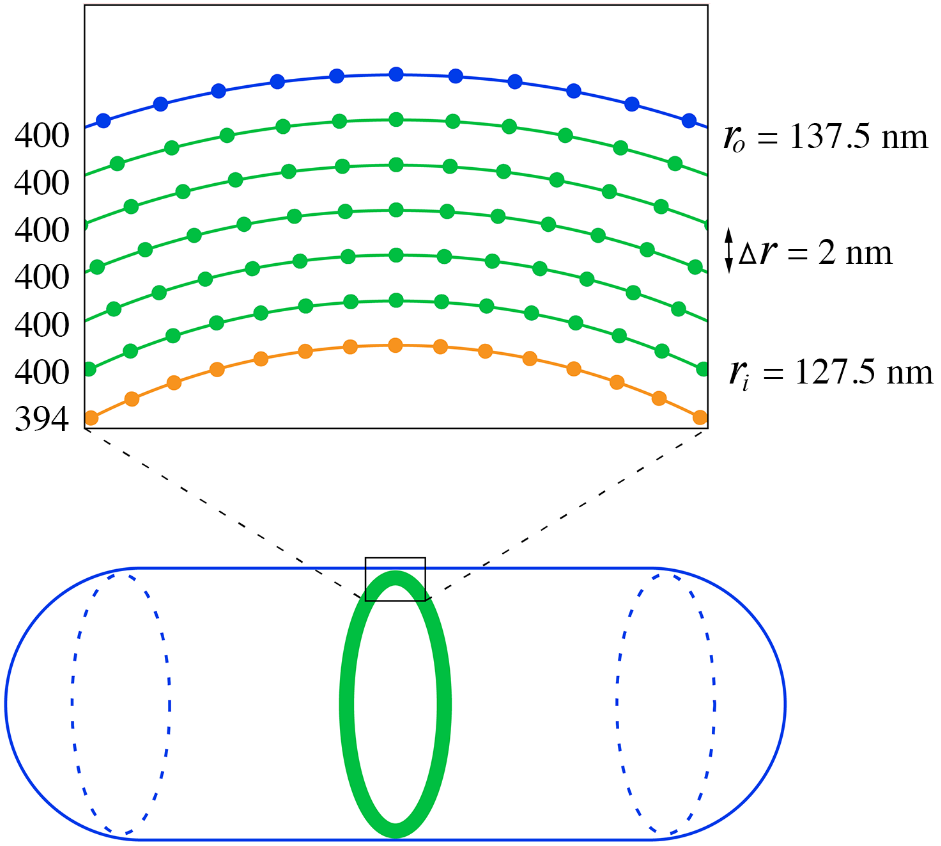

Supplementary Figure 6: Schematic of the make-before-break mechanism with a multi-layered septum. Each hoop on the existing PG layer (blue) is composed of 400 tetrasaccharides and expanded under turgor pressure to a radius r_o = 137.5 nm. In a multi-layered septum with adjacent layers separated by a 2-nm distance, the hoops on the first five layers (green) would also be composed of 400 tetrasaccharides each. Since the fifth layer has a radius of r_i = 127.5 nm, it is in a relaxed state. Therefore, the sixth layer (orange) would consist of 394-tetrasaccharide hoops.
