# Supplementary material for: Simulations suggest a constrictive force is required for Gram-negative bacterial cell division
Source: Nat Commun. 2019 Mar 19;10:1259. doi: 10.1038/s41467-019-09264-0 (PMC6425016; doi:10.1038/s41467-019-09264-0)
Supplement: Supplementary file 3 — Description of Additional Supplementary Files [file 41467_2019_9264_MOESM3_ESM.pdf]

Description of Additional Supplementary Files

File Name: Supplementary Movie 1
Description: This movie summarizes our simulations of leading hypotheses in the field about what drives Gram-negative bacterial cell division, including (1) simply reorganizing the cell wall synthesis machinery to the midcell, (2) the presence of a constrictive force, (3) a make-before-break mechanism of cell wall remodeling, and (4) the make-before-break mechanism in the presence of a constrictive force.

File Name: Supplementary Software 1
Description: The software package is contained in the file demo.zip which includes readme.txt, Remodeler2.f90, Mods.f90, Vars.f90, config0000.dat, coor0000.dat, restart.dat, paras.info
